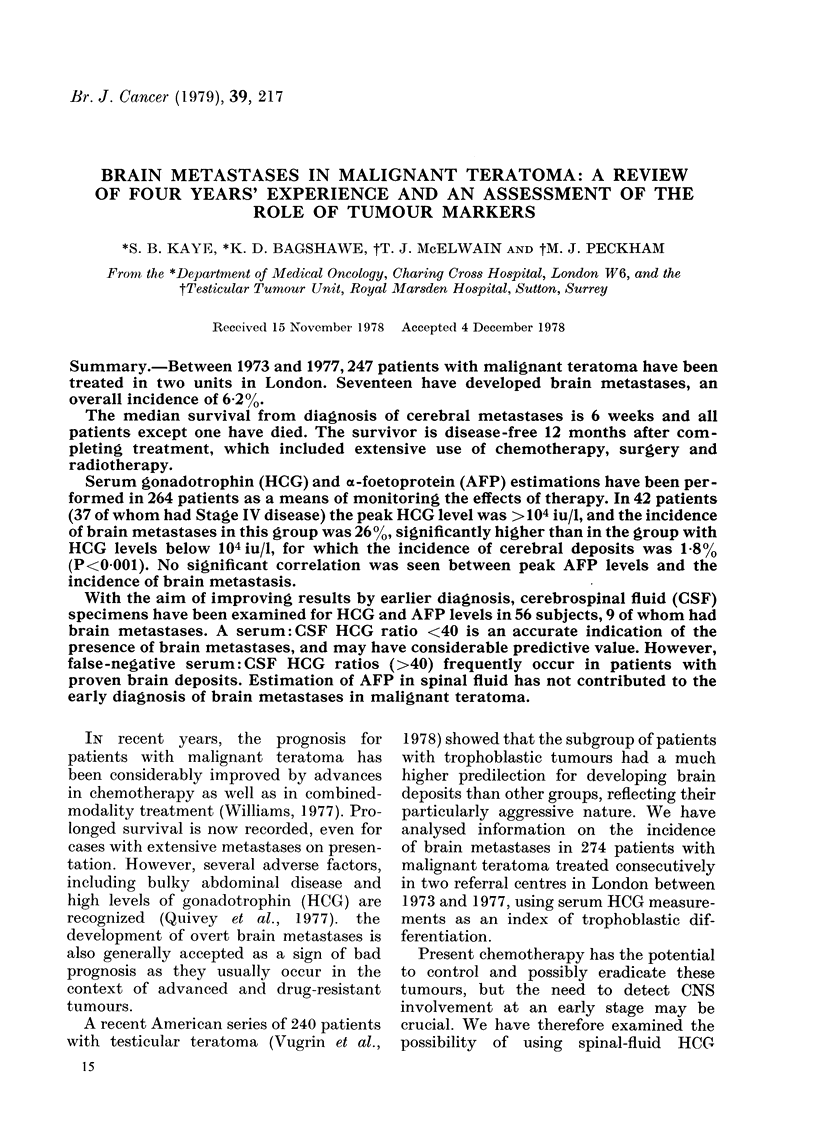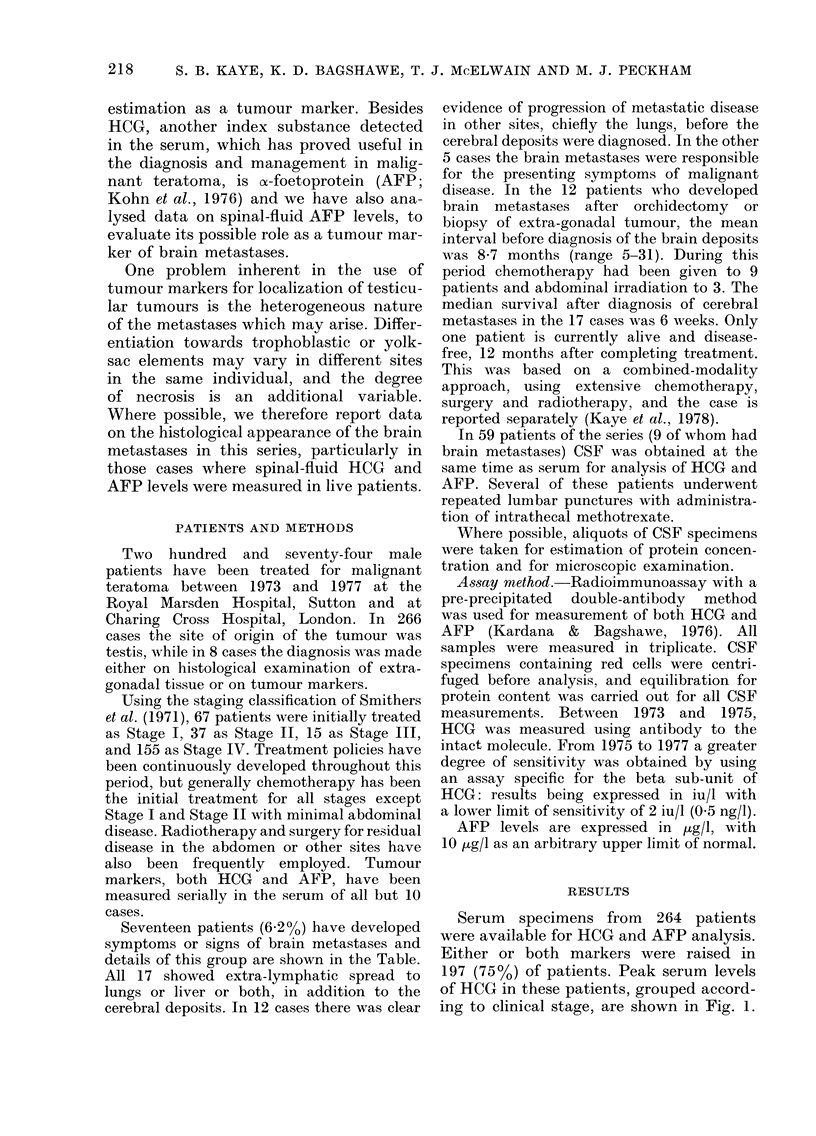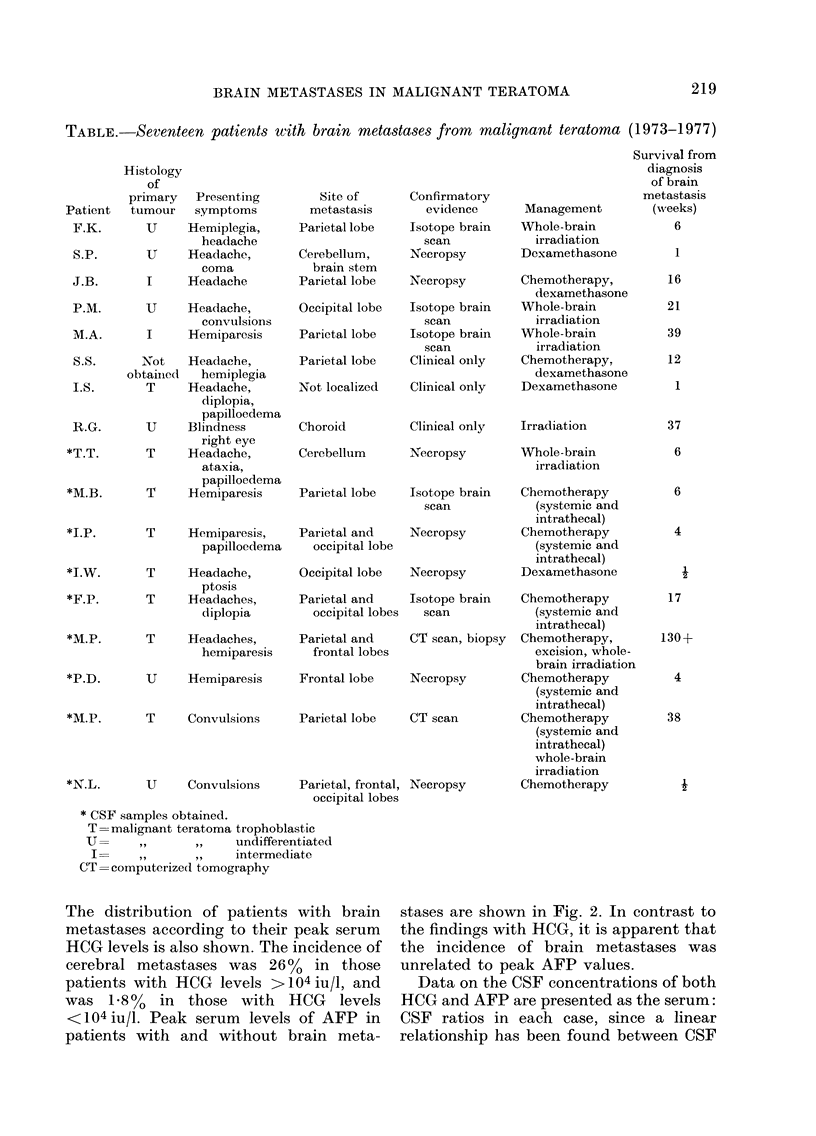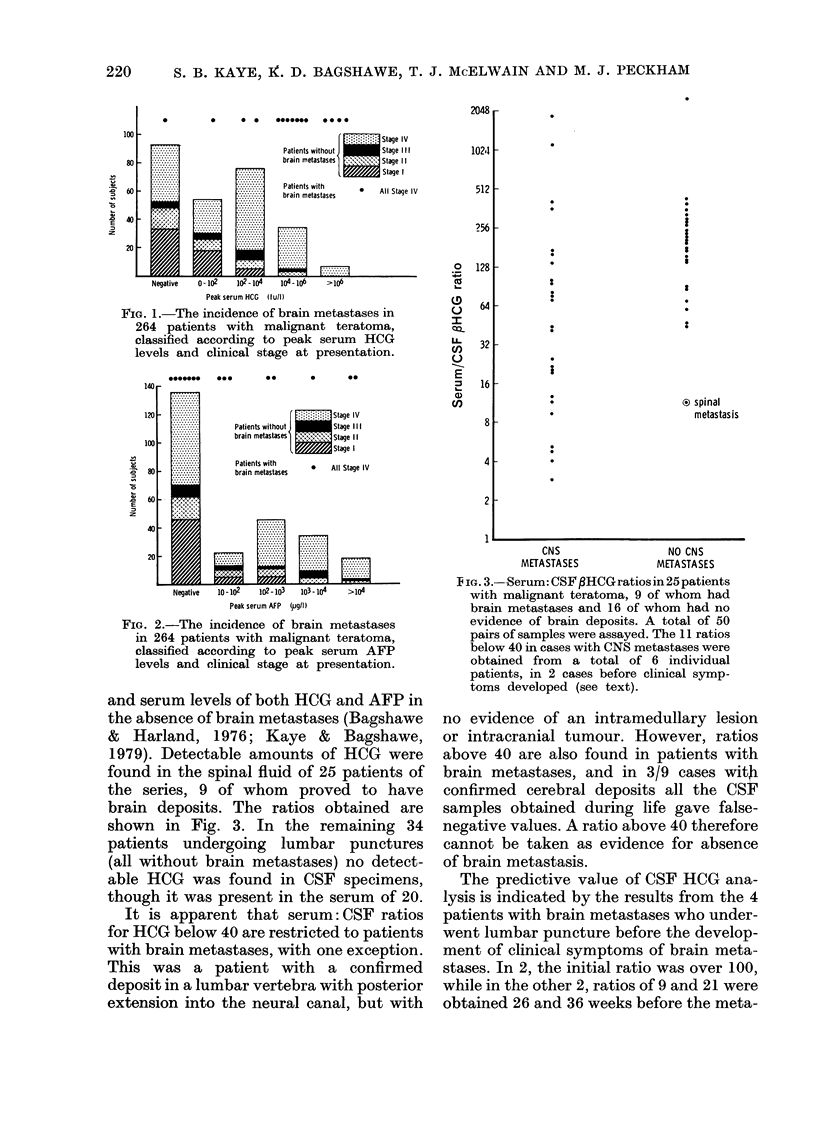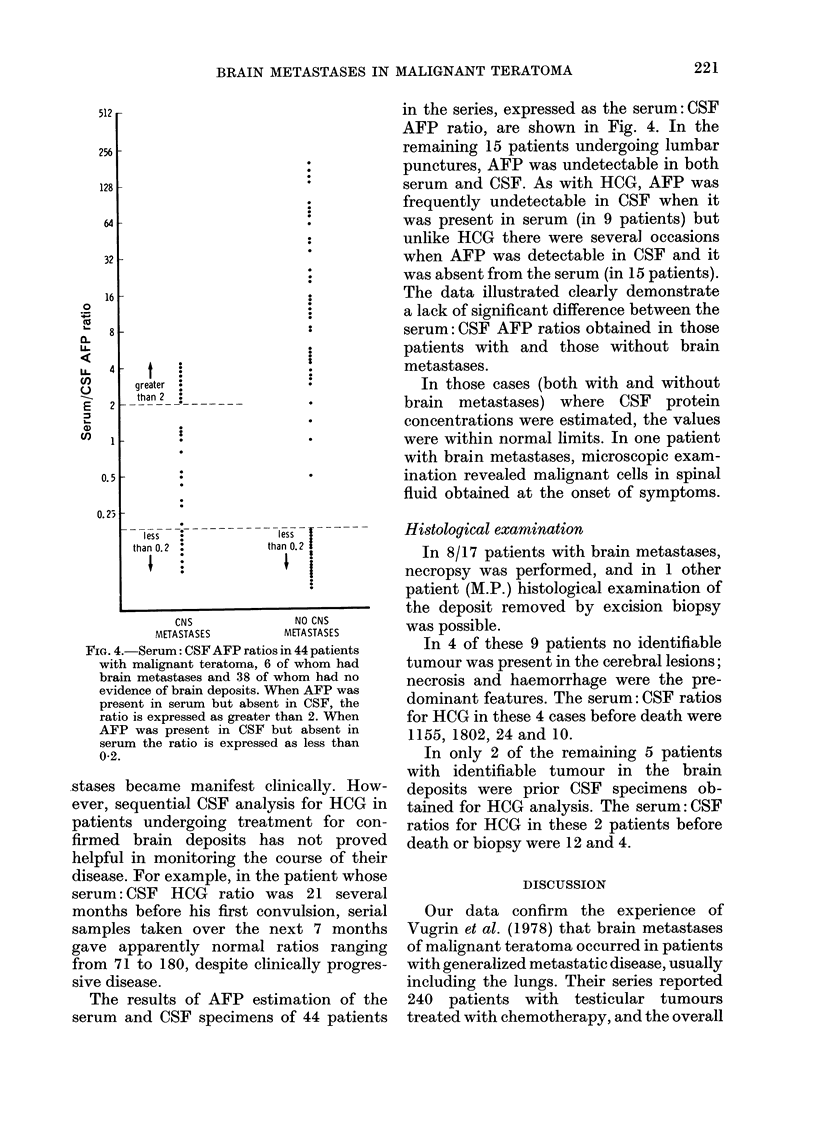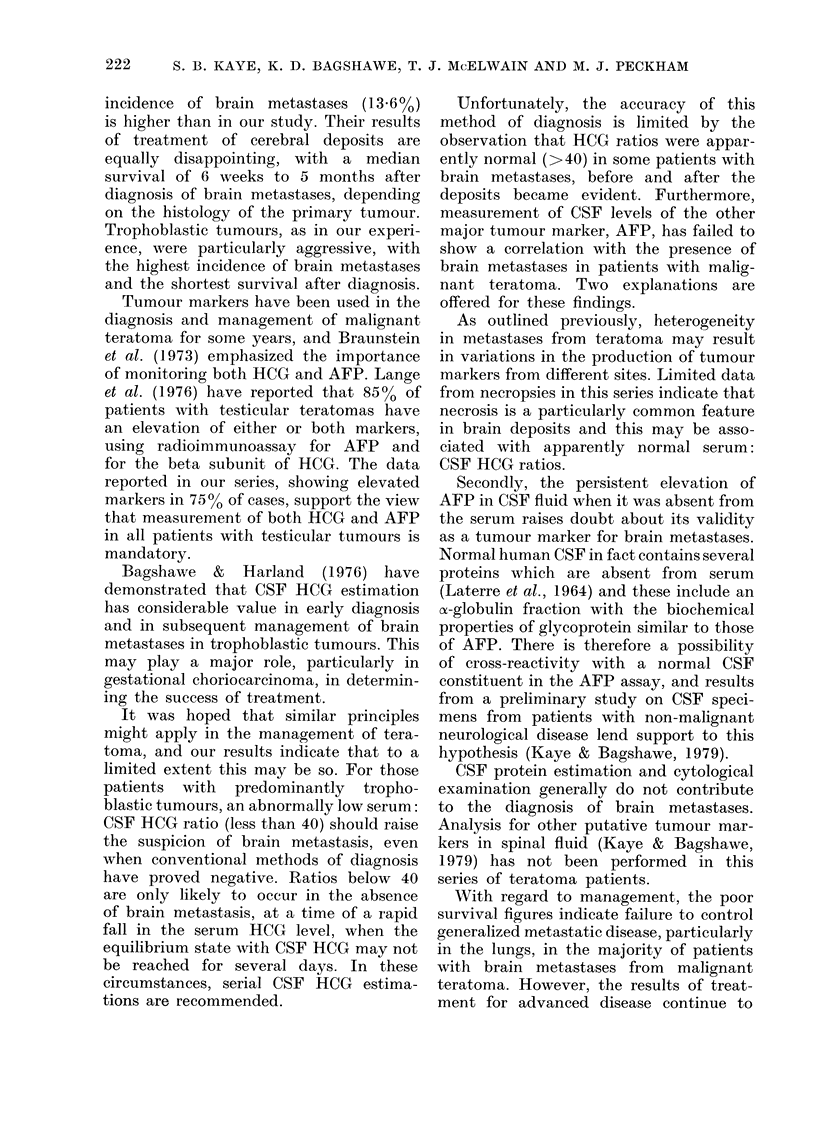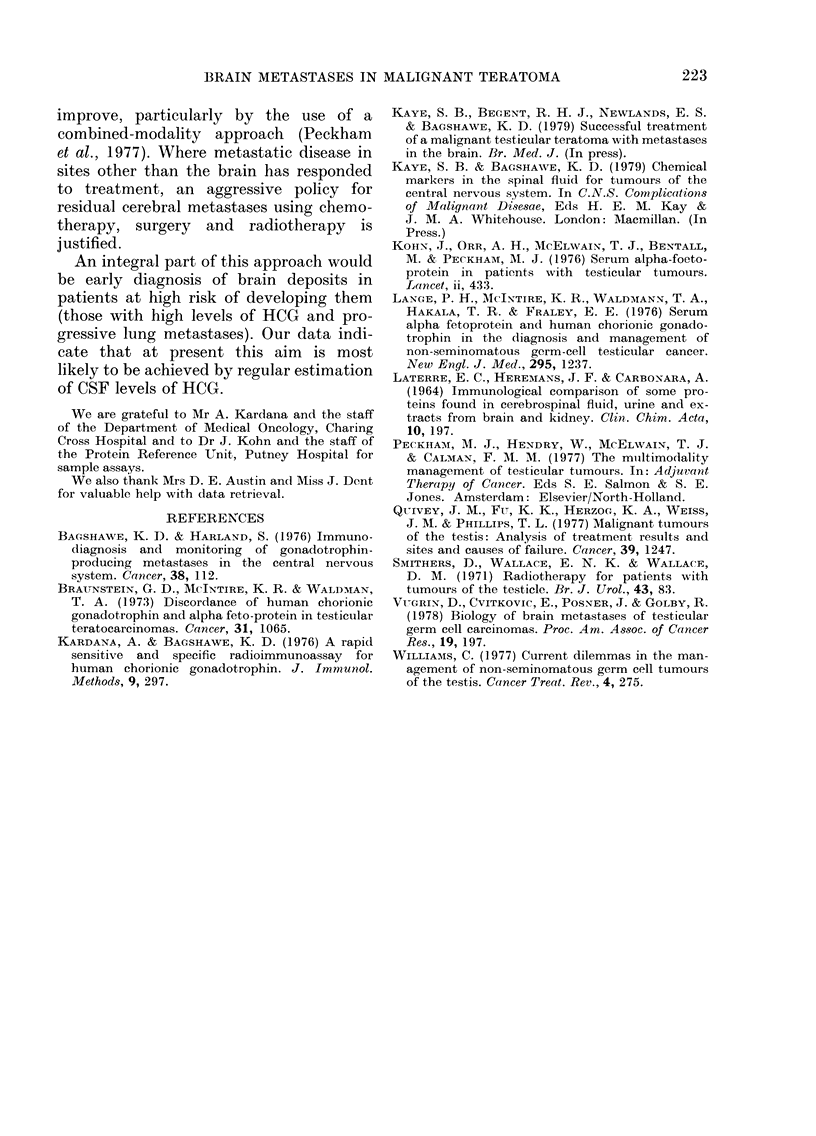# Brain metastases in malignant teratoma: a review of four years' experience and an assessment of the role of tumour markers.

**DOI:** 10.1038/bjc.1979.44

**Published:** 1979-03

**Authors:** S. B. Kaye, K. D. Bagshawe, T. J. McElwain, M. J. Peckham

## Abstract

Between 1973 and 1977, 247 patients with malignant teratoma have been treated in two units in London. Seventeen have developed brain metastases, an overall incidence of 6.2%. The median survival from diagnosis of cerebral metastases is 6 weeks and all patients except one have died. The survivor is disease-free 12 months after completing treatment, which included extensive use of chemotherapy, surgery and radiotherapy. Serum gonadotrophin (HCG) and alpha-foetoprotein (AFP) estimations have been performed in 264 patients as a means of monitoring the effects of therapy. In 42 patients (37 of whom had Stage IV disease) the peak HCG level was greater than 10(4) iu/l, and the incidence of brain metastases in this group was 26%, significantly higher than in the group with HCG levels below 10(4) iu/l, for which the incidence of cerebral deposits was 1.8% (P less than 0.0001). No significant correlation was seen between peak AFP levels and the incidence of brain metastasis. With the aim of improving results by earlier diagnosis, cerebrospinal fluid (CSF) specimens have been examined for HCG and AFP levels in 56 subjects, 9 of whom had brain metastases. A serum: CSF HCG ratio less than 40 is an accurate indication of the presence of brain metastases, and may have considerable predictive value. However, false-negative serum: CSF HCG rations (greater than 40) frequently occur in patients with proven brain deposits. Estimation of AFP in spinal fluid has not contributed to the early diagnosis of brain metastases in malignant teratoma.


*Br. J. Cancer* (1979), **39**, 217

### BRAIN METASTASES IN MALIGNANT TERATOMA: A REVIEW OF FOUR YEARS' EXPERIENCE AND AN ASSESSMENT OF THE ROLE OF TUMOUR MARKERS

*S. B. KAYE, *K. D. BAGSHAWE, †T. J. McELWAIN AND †M. J. PECKHAM

*From the *Department of Medical Oncology, Charing Cross Hospital, London W6, and the †Testicular Tumour Unit, Royal Marsden Hospital, Sutton, Surrey*

Received 15 November 1978 Accepted 4 December 1978

**Summary.—Between 1973 and 1977, 247 patients with malignant teratoma have been treated in two units in London. Seventeen have developed brain metastases, an overall incidence of 6·2%.**

**The median survival from diagnosis of cerebral metastases is 6 weeks and all patients except one have died. The survivor is disease-free 12 months after completing treatment, which included extensive use of chemotherapy, surgery and radiotherapy.**

**Serum gonadotrophin (HCG) and α-foetoprotein (AFP) estimations have been performed in 264 patients as a means of monitoring the effects of therapy. In 42 patients (37 of whom had Stage IV disease) the peak HCG level was $>10^4$ iu/l, and the incidence of brain metastases in this group was 26%, significantly higher than in the group with HCG levels below $10^4$ iu/l, for which the incidence of cerebral deposits was 1·8% ($P<0{\cdot}001$). No significant correlation was seen between peak AFP levels and the incidence of brain metastasis.**

**With the aim of improving results by earlier diagnosis, cerebrospinal fluid (CSF) specimens have been examined for HCG and AFP levels in 56 subjects, 9 of whom had brain metastases. A serum:CSF HCG ratio <40 is an accurate indication of the presence of brain metastases, and may have considerable predictive value. However, false-negative serum:CSF HCG ratios (>40) frequently occur in patients with proven brain deposits. Estimation of AFP in spinal fluid has not contributed to the early diagnosis of brain metastases in malignant teratoma.**

In recent years, the prognosis for patients with malignant teratoma has been considerably improved by advances in chemotherapy as well as in combined-modality treatment (Williams, 1977). Prolonged survival is now recorded, even for cases with extensive metastases on presentation. However, several adverse factors, including bulky abdominal disease and high levels of gonadotrophin (HCG) are recognized (Quivey *et al.*, 1977). the development of overt brain metastases is also generally accepted as a sign of bad prognosis as they usually occur in the context of advanced and drug-resistant tumours.

A recent American series of 240 patients with testicular teratoma (Vugrin *et al.*, 1978) showed that the subgroup of patients with trophoblastic tumours had a much higher predilection for developing brain deposits than other groups, reflecting their particularly aggressive nature. We have analysed information on the incidence of brain metastases in 274 patients with malignant teratoma treated consecutively in two referral centres in London between 1973 and 1977, using serum HCG measurements as an index of trophoblastic differentiation.

Present chemotherapy has the potential to control and possibly eradicate these tumours, but the need to detect CNS involvement at an early stage may be crucial. We have therefore examined the possibility of using spinal-fluid HCG

estimation as a tumour marker. Besides HCG, another index substance detected in the serum, which has proved useful in the diagnosis and management in malignant teratoma, is α-foetoprotein (AFP; Kohn *et al.*, 1976) and we have also analysed data on spinal-fluid AFP levels, to evaluate its possible role as a tumour marker of brain metastases.

One problem inherent in the use of tumour markers for localization of testicular tumours is the heterogeneous nature of the metastases which may arise. Differentiation towards trophoblastic or yolk-sac elements may vary in different sites in the same individual, and the degree of necrosis is an additional variable. Where possible, we therefore report data on the histological appearance of the brain metastases in this series, particularly in those cases where spinal-fluid HCG and AFP levels were measured in live patients.

### PATIENTS AND METHODS

Two hundred and seventy-four male patients have been treated for malignant teratoma between 1973 and 1977 at the Royal Marsden Hospital, Sutton and at Charing Cross Hospital, London. In 266 cases the site of origin of the tumour was testis, while in 8 cases the diagnosis was made either on histological examination of extra-gonadal tissue or on tumour markers.

Using the staging classification of Smithers *et al.* (1971), 67 patients were initially treated as Stage I, 37 as Stage II, 15 as Stage III, and 155 as Stage IV. Treatment policies have been continuously developed throughout this period, but generally chemotherapy has been the initial treatment for all stages except Stage I and Stage II with minimal abdominal disease. Radiotherapy and surgery for residual disease in the abdomen or other sites have also been frequently employed. Tumour markers, both HCG and AFP, have been measured serially in the serum of all but 10 cases.

Seventeen patients (6·2%) have developed symptoms or signs of brain metastases and details of this group are shown in the Table. All 17 showed extra-lymphatic spread to lungs or liver or both, in addition to the cerebral deposits. In 12 cases there was clear evidence of progression of metastatic disease in other sites, chiefly the lungs, before the cerebral deposits were diagnosed. In the other 5 cases the brain metastases were responsible for the presenting symptoms of malignant disease. In the 12 patients who developed brain metastases after orchidectomy or biopsy of extra-gonadal tumour, the mean interval before diagnosis of the brain deposits was 8·7 months (range 5–31). During this period chemotherapy had been given to 9 patients and abdominal irradiation to 3. The median survival after diagnosis of cerebral metastases in the 17 cases was 6 weeks. Only one patient is currently alive and disease-free, 12 months after completing treatment. This was based on a combined-modality approach, using extensive chemotherapy, surgery and radiotherapy, and the case is reported separately (Kaye *et al.*, 1978).

In 59 patients of the series (9 of whom had brain metastases) CSF was obtained at the same time as serum for analysis of HCG and AFP. Several of these patients underwent repeated lumbar punctures with administration of intrathecal methotrexate.

Where possible, aliquots of CSF specimens were taken for estimation of protein concentration and for microscopic examination.

*Assay method.*—Radioimmunoassay with a pre-precipitated double-antibody method was used for measurement of both HCG and AFP (Kardana & Bagshawe, 1976). All samples were measured in triplicate. CSF specimens containing red cells were centrifuged before analysis, and equilibration for protein content was carried out for all CSF measurements. Between 1973 and 1975, HCG was measured using antibody to the intact molecule. From 1975 to 1977 a greater degree of sensitivity was obtained by using an assay specific for the beta sub-unit of HCG: results being expressed in iu/l with a lower limit of sensitivity of 2 iu/l (0·5 ng/l).

AFP levels are expressed in μg/l, with 10 μg/l as an arbitrary upper limit of normal.

### RESULTS

Serum specimens from 264 patients were available for HCG and AFP analysis. Either or both markers were raised in 197 (75%) of patients. Peak serum levels of HCG in these patients, grouped according to clinical stage, are shown in Fig. 1.

TABLE.—*Seventeen patients with brain metastases from malignant teratoma* (1973–1977)

| Patient | Histology of primary tumour | Presenting symptoms | Site of metastasis | Confirmatory evidence | Management | Survival from diagnosis of brain metastasis (weeks) |
|---|---|---|---|---|---|---|
| F.K. | U | Hemiplegia, headache | Parietal lobe | Isotope brain scan | Whole-brain irradiation | 6 |
| S.P. | U | Headache, coma | Cerebellum, brain stem | Necropsy | Dexamethasone | 1 |
| J.B. | I | Headache | Parietal lobe | Necropsy | Chemotherapy, dexamethasone | 16 |
| P.M. | U | Headache, convulsions | Occipital lobe | Isotope brain scan | Whole-brain irradiation | 21 |
| M.A. | I | Hemiparesis | Parietal lobe | Isotope brain scan | Whole-brain irradiation | 39 |
| S.S. | Not obtained | Headache, hemiplegia | Parietal lobe | Clinical only | Chemotherapy, dexamethasone | 12 |
| I.S. | T | Headache, diplopia, papilloedema | Not localized | Clinical only | Dexamethasone | 1 |
| R.G. | U | Blindness right eye | Choroid | Clinical only | Irradiation | 37 |
| *T.T. | T | Headache, ataxia, papilloedema | Cerebellum | Necropsy | Whole-brain irradiation | 6 |
| *M.B. | T | Hemiparesis | Parietal lobe | Isotope brain scan | Chemotherapy (systemic and intrathecal) | 6 |
| *I.P. | T | Hemiparesis, papilloedema | Parietal and occipital lobe | Necropsy | Chemotherapy (systemic and intrathecal) | 4 |
| *I.W. | T | Headache, ptosis | Occipital lobe | Necropsy | Dexamethasone | $\frac{1}{2}$ |
| *F.P. | T | Headaches, diplopia | Parietal and occipital lobes | Isotope brain scan | Chemotherapy (systemic and intrathecal) | 17 |
| *M.P. | T | Headaches, hemiparesis | Parietal and frontal lobes | CT scan, biopsy | Chemotherapy, excision, whole-brain irradiation | 130+ |
| *P.D. | U | Hemiparesis | Frontal lobe | Necropsy | Chemotherapy (systemic and intrathecal) | 4 |
| *M.P. | T | Convulsions | Parietal lobe | CT scan | Chemotherapy (systemic and intrathecal) whole-brain irradiation | 38 |
| *N.L. | U | Convulsions | Parietal, frontal, occipital lobes | Necropsy | Chemotherapy | $\frac{1}{2}$ |

* CSF samples obtained.
T = malignant teratoma trophoblastic
U = ,, ,, undifferentiated
I = ,, ,, intermediate
CT = computerized tomography

The distribution of patients with brain metastases according to their peak serum HCG levels is also shown. The incidence of cerebral metastases was 26% in those patients with HCG levels $>10^4$ iu/l, and was 1·8% in those with HCG levels $<10^4$ iu/l. Peak serum levels of AFP in patients with and without brain metastases are shown in Fig. 2. In contrast to the findings with HCG, it is apparent that the incidence of brain metastases was unrelated to peak AFP values.

Data on the CSF concentrations of both HCG and AFP are presented as the serum: CSF ratios in each case, since a linear relationship has been found between CSF

FIG. 1.—The incidence of brain metastases in 264 patients with malignant teratoma, classified according to peak serum HCG levels and clinical stage at presentation.

FIG. 2.—The incidence of brain metastases in 264 patients with malignant teratoma, classified according to peak serum AFP levels and clinical stage at presentation.

FIG. 3.—Serum: CSF βHCG ratios in 25 patients with malignant teratoma, 9 of whom had brain metastases and 16 of whom had no evidence of brain deposits. A total of 50 pairs of samples were assayed. The 11 ratios below 40 in cases with CNS metastases were obtained from a total of 6 individual patients, in 2 cases before clinical symptoms developed (see text).

and serum levels of both HCG and AFP in the absence of brain metastases (Bagshawe & Harland, 1976; Kaye & Bagshawe, 1979). Detectable amounts of HCG were found in the spinal fluid of 25 patients of the series, 9 of whom proved to have brain deposits. The ratios obtained are shown in Fig. 3. In the remaining 34 patients undergoing lumbar punctures (all without brain metastases) no detectable HCG was found in CSF specimens, though it was present in the serum of 20.

It is apparent that serum: CSF ratios for HCG below 40 are restricted to patients with brain metastases, with one exception. This was a patient with a confirmed deposit in a lumbar vertebra with posterior extension into the neural canal, but with no evidence of an intramedullary lesion or intracranial tumour. However, ratios above 40 are also found in patients with brain metastases, and in 3/9 cases with confirmed cerebral deposits all the CSF samples obtained during life gave false-negative values. A ratio above 40 therefore cannot be taken as evidence for absence of brain metastasis.

The predictive value of CSF HCG analysis is indicated by the results from the 4 patients with brain metastases who underwent lumbar puncture before the development of clinical symptoms of brain metastases. In 2, the initial ratio was over 100, while in the other 2, ratios of 9 and 21 were obtained 26 and 36 weeks before the meta-

FIG. 4.—Serum : CSF AFP ratios in 44 patients with malignant teratoma, 6 of whom had brain metastases and 38 of whom had no evidence of brain deposits. When AFP was present in serum but absent in CSF, the ratio is expressed as greater than 2. When AFP was present in CSF but absent in serum the ratio is expressed as less than 0·2.

stases became manifest clinically. However, sequential CSF analysis for HCG in patients undergoing treatment for confirmed brain deposits has not proved helpful in monitoring the course of their disease. For example, in the patient whose serum:CSF HCG ratio was 21 several months before his first convulsion, serial samples taken over the next 7 months gave apparently normal ratios ranging from 71 to 180, despite clinically progressive disease.

The results of AFP estimation of the serum and CSF specimens of 44 patients in the series, expressed as the serum:CSF AFP ratio, are shown in Fig. 4. In the remaining 15 patients undergoing lumbar punctures, AFP was undetectable in both serum and CSF. As with HCG, AFP was frequently undetectable in CSF when it was present in serum (in 9 patients) but unlike HCG there were several occasions when AFP was detectable in CSF and it was absent from the serum (in 15 patients). The data illustrated clearly demonstrate a lack of significant difference between the serum:CSF AFP ratios obtained in those patients with and those without brain metastases.

In those cases (both with and without brain metastases) where CSF protein concentrations were estimated, the values were within normal limits. In one patient with brain metastases, microscopic examination revealed malignant cells in spinal fluid obtained at the onset of symptoms.

*Histological examination*

In 8/17 patients with brain metastases, necropsy was performed, and in 1 other patient (M.P.) histological examination of the deposit removed by excision biopsy was possible.

In 4 of these 9 patients no identifiable tumour was present in the cerebral lesions; necrosis and haemorrhage were the predominant features. The serum: CSF ratios for HCG in these 4 cases before death were 1155, 1802, 24 and 10.

In only 2 of the remaining 5 patients with identifiable tumour in the brain deposits were prior CSF specimens obtained for HCG analysis. The serum:CSF ratios for HCG in these 2 patients before death or biopsy were 12 and 4.

### DISCUSSION

Our data confirm the experience of Vugrin *et al.* (1978) that brain metastases of malignant teratoma occurred in patients with generalized metastatic disease, usually including the lungs. Their series reported 240 patients with testicular tumours treated with chemotherapy, and the overall incidence of brain metastases (13·6%) is higher than in our study. Their results of treatment of cerebral deposits are equally disappointing, with a median survival of 6 weeks to 5 months after diagnosis of brain metastases, depending on the histology of the primary tumour. Trophoblastic tumours, as in our experience, were particularly aggressive, with the highest incidence of brain metastases and the shortest survival after diagnosis.

Tumour markers have been used in the diagnosis and management of malignant teratoma for some years, and Braunstein *et al.* (1973) emphasized the importance of monitoring both HCG and AFP. Lange *et al.* (1976) have reported that 85% of patients with testicular teratomas have an elevation of either or both markers, using radioimmunoassay for AFP and for the beta subunit of HCG. The data reported in our series, showing elevated markers in 75% of cases, support the view that measurement of both HCG and AFP in all patients with testicular tumours is mandatory.

Bagshawe & Harland (1976) have demonstrated that CSF HCG estimation has considerable value in early diagnosis and in subsequent management of brain metastases in trophoblastic tumours. This may play a major role, particularly in gestational choriocarcinoma, in determining the success of treatment.

It was hoped that similar principles might apply in the management of teratoma, and our results indicate that to a limited extent this may be so. For those patients with predominantly trophoblastic tumours, an abnormally low serum: CSF HCG ratio (less than 40) should raise the suspicion of brain metastasis, even when conventional methods of diagnosis have proved negative. Ratios below 40 are only likely to occur in the absence of brain metastasis, at a time of a rapid fall in the serum HCG level, when the equilibrium state with CSF HCG may not be reached for several days. In these circumstances, serial CSF HCG estimations are recommended.

Unfortunately, the accuracy of this method of diagnosis is limited by the observation that HCG ratios were apparently normal (>40) in some patients with brain metastases, before and after the deposits became evident. Furthermore, measurement of CSF levels of the other major tumour marker, AFP, has failed to show a correlation with the presence of brain metastases in patients with malignant teratoma. Two explanations are offered for these findings.

As outlined previously, heterogeneity in metastases from teratoma may result in variations in the production of tumour markers from different sites. Limited data from necropsies in this series indicate that necrosis is a particularly common feature in brain deposits and this may be associated with apparently normal serum: CSF HCG ratios.

Secondly, the persistent elevation of AFP in CSF fluid when it was absent from the serum raises doubt about its validity as a tumour marker for brain metastases. Normal human CSF in fact contains several proteins which are absent from serum (Laterre *et al.*, 1964) and these include an α-globulin fraction with the biochemical properties of glycoprotein similar to those of AFP. There is therefore a possibility of cross-reactivity with a normal CSF constituent in the AFP assay, and results from a preliminary study on CSF specimens from patients with non-malignant neurological disease lend support to this hypothesis (Kaye & Bagshawe, 1979).

CSF protein estimation and cytological examination generally do not contribute to the diagnosis of brain metastases. Analysis for other putative tumour markers in spinal fluid (Kaye & Bagshawe, 1979) has not been performed in this series of teratoma patients.

With regard to management, the poor survival figures indicate failure to control generalized metastatic disease, particularly in the lungs, in the majority of patients with brain metastases from malignant teratoma. However, the results of treatment for advanced disease continue to improve, particularly by the use of a combined-modality approach (Peckham *et al.*, 1977). Where metastatic disease in sites other than the brain has responded to treatment, an aggressive policy for residual cerebral metastases using chemotherapy, surgery and radiotherapy is justified.

An integral part of this approach would be early diagnosis of brain deposits in patients at high risk of developing them (those with high levels of HCG and progressive lung metastases). Our data indicate that at present this aim is most likely to be achieved by regular estimation of CSF levels of HCG.

We are grateful to Mr A. Kardana and the staff of the Department of Medical Oncology, Charing Cross Hospital and to Dr J. Kohn and the staff of the Protein Reference Unit, Putney Hospital for sample assays.

We also thank Mrs D. E. Austin and Miss J. Dent for valuable help with data retrieval.

### REFERENCES

BAGSHAWE, K. D. & HARLAND, S. (1976) Immunodiagnosis and monitoring of gonadotrophin-producing metastases in the central nervous system. *Cancer*, **38**, 112.

BRAUNSTEIN, G. D., MCINTIRE, K. R. & WALDMAN, T. A. (1973) Discordance of human chorionic gonadotrophin and alpha feto-protein in testicular teratocarcinomas. *Cancer*, **31**, 1065.

KARDANA, A. & BAGSHAWE, K. D. (1976) A rapid sensitive and specific radioimmunoassay for human chorionic gonadotrophin. *J. Immunol. Methods*, **9**, 297.

KAYE, S. B., BEGENT, R. H. J., NEWLANDS, E. S. & BAGSHAWE, K. D. (1979) Successful treatment of a malignant testicular teratoma with metastases in the brain. *Br. Med. J.* (In press).

KAYE, S. B. & BAGSHAWE, K. D. (1979) Chemical markers in the spinal fluid for tumours of the central nervous system. In *C.N.S. Complications of Malignant Disesae*, Eds H. E. M. Kay & J. M. A. Whitehouse. London: Macmillan. (In Press.)

KOHN, J., ORR, A. H., MCELWAIN, T. J., BENTALL, M. & PECKHAM, M. J. (1976) Serum alpha-foeto-protein in patients with testicular tumours. *Lancet*, ii, 433.

LANGE, P. H., MCINTIRE, K. R., WALDMANN, T. A., HAKALA, T. R. & FRALEY, E. E. (1976) Serum alpha fetoprotein and human chorionic gonadotrophin in the diagnosis and management of non-seminomatous germ-cell testicular cancer. *New Engl. J. Med.*, **295**, 1237.

LATERRE, E. C., HEREMANS, J. F. & CARBONARA, A. (1964) Immunological comparison of some proteins found in cerebrospinal fluid, urine and extracts from brain and kidney. *Clin. Chim. Acta*, **10**, 197.

PECKHAM, M. J., HENDRY, W., MCELWAIN, T. J. & CALMAN, F. M. M. (1977) The multimodality management of testicular tumours. In: *Adjuvant Therapy of Cancer*. Eds S. E. Salmon & S. E. Jones. Amsterdam: Elsevier/North-Holland.

QUIVEY, J. M., FU, K. K., HERZOG, K. A., WEISS, J. M. & PHILLIPS, T. L. (1977) Malignant tumours of the testis: Analysis of treatment results and sites and causes of failure. *Cancer*, **39**, 1247.

SMITHERS, D., WALLACE, E. N. K. & WALLACE, D. M. (1971) Radiotherapy for patients with tumours of the testicle. *Br. J. Urol.*, **43**, 83.

VUGRIN, D., CVITKOVIC, E., POSNER, J. & GOLBY, R. (1978) Biology of brain metastases of testicular germ cell carcinomas. *Proc. Am. Assoc. of Cancer Res.*, **19**, 197.

WILLIAMS, C. (1977) Current dilemmas in the management of non-seminomatous germ cell tumours of the testis. *Cancer Treat. Rev.*, **4**, 275.